# Hemoglobin-to-red blood cell distribution width ratio: a new insight into cognitive protection for obese individuals

**DOI:** 10.3389/fmed.2025.1625542

**Published:** 2025-09-26

**Authors:** Ruikai Xu, Zelin Wu, Zhonghua Liu

**Affiliations:** Department of Rehabilitation, Zhongshan People’s Hospital, Zhongshan, Guangdong, China

**Keywords:** hemoglobin, red blood cell distribution width ratio, cognitive function, obese, NHANES

## Abstract

**Background and Objective:**

Aging and obesity are recognized as risk factors for cognitive decline. Hemoglobin (Hb) reflects oxygen supply capacity, while red blood cell distribution width (RDW) reflects levels of inflammation and oxidative stress. The hemoglobin-to-red blood cell distribution width ratio (HRR), by integrating the core physiological functions of Hb and RDW, can more comprehensively reflect the common mechanisms affecting aging, obesity, and cognitive function. The objective of this research was to explore the link between the HRR and cognitive performance among the obese population.

**Methods:**

This cross-sectional study used data from the National Health and Nutrition Examination Survey (NHANES) and employed multiple regression analysis, smooth curve fitting, and subgroup analysis to investigate the relationship between HRR and cognitive function.

**Results:**

1,055 obese individuals aged ≥60 years participated in the study. After adjusting for covariates, HRR was significantly positively correlated with DSST scores (β = 14.45; 95% CI, 7.55–21.35) and total cognitive Z-scores (β = 1.53; 95% CI, 0.40–2.67). HRR was significantly negatively correlated with low cognitive function as assessed by DSST (OR = 0.04; 95% CI, 0.01–0.23). Compared to individuals with lower education levels, those with higher educational backgrounds showed a more pronounced positive correlation between HRR and DSST scores.

**Conclusion:**

Maintaining a higher HRR may be an important strategy for protecting cognitive function in obese individuals aged ≥60 years.

## 1 Background

As humans age, there is often a decline in cognitive function, which significantly increases the risk of developing mild cognitive impairment and eventually dementia (1). In patients with dementia, cognitive impairment severely impacts quality of life and the ability to live independently (2).

Cognitive impairment has become a major public health issue (3). By the middle of the 21st century, it is projected that the number of individuals with cognitive impairment in the United States will exceed 21 million, while the global number of dementia patients will surpass 150 million (3, 4). This growing trend will impose substantial burdens on individuals, society, and the economy (5).

The relationship between obesity and cognitive impairment is particularly significant (6). Obesity increases the risk of developing Alzheimer’s disease, stroke-related dementia (6, 7). Obesity increases cognitive impairment risk via multiple mechanisms: High-fat diets trigger brain inflammation in areas like the hypothalamus and hippocampus, releasing inflammatory factors that damage neural structures (8–10). This process also causes lipid peroxidation, harming the blood-brain barrier (11). Insulin resistance in the brain impairs glucose use and synaptic function, while leptin resistance and ghrelin imbalance disrupt appetite control and reinforce rewards system sensitivity to fatty foods (12, 13). These mechanisms together reduce gray matter volume in key brain regions, degrade white matter integrity, decrease blood flow, and weaken network connectivity, ultimately worsening cognitive decline and dementia risk (14, 15).

As a core factor maintaining the homeostasis of cerebral oxygen supply, hemoglobin (Hb) participates in the regulation of cognitive function by regulating oxygen metabolism (16). When Hb levels decrease, the oxygen delivery through cerebral blood flow fails to match metabolic demands, which can induce functional impairment of brain cells and accelerate the progression of cognitive decline (16, 17). Red blood cell distribution width (RDW) is significantly correlated with systemic inflammatory response and oxidative stress levels, and these mechanisms are the common core pathophysiological pathways driving aging, obesity, and cognitive decline (18, 19). By integrating the oxygen supply regulatory function of Hb and the inflammation and oxidative stress signals reflected by RDW, hemoglobin-to-red blood cell distribution width ratio (HRR) constructs a multi-dimensional association mechanism with aging, obesity, and cognitive function. As a composite biomarker integrating the core physiological functions of Hb and RDW, HRR can more comprehensively reflect the common mechanisms affecting aging, obesity, and cognitive function including inflammation, oxidative stress, abnormal oxygen metabolism, and nerve damage, thereby overcoming the limitation that a single biomarker can only reflect local pathophysiological processes (14). The HRR has shown promise in predicting various diseases, including depression (20), coronary artery disease (21), stroke (22), osteoporosis (23), and metastatic kidney cancer (24) in recent years.

Notably, research on the HRR and cognitive function remains scarce, particularly among obese individuals aged ≥60 years. We hypothesize that in obese populations, higher HRR correlates with better cognitive performance, independent of confounders like age and comorbidities, potentially mediated by improved cerebral oxygenation and reduced systemic inflammation. This study explores HRR’s association with cognitive function in this group, examining whether elevated HRR acts as a cognitive protective factor–offering new insights into cognitive protection for obese older adults.

## 2 Materials and methods

### 2.1 Study population

**FIGURE 1:**
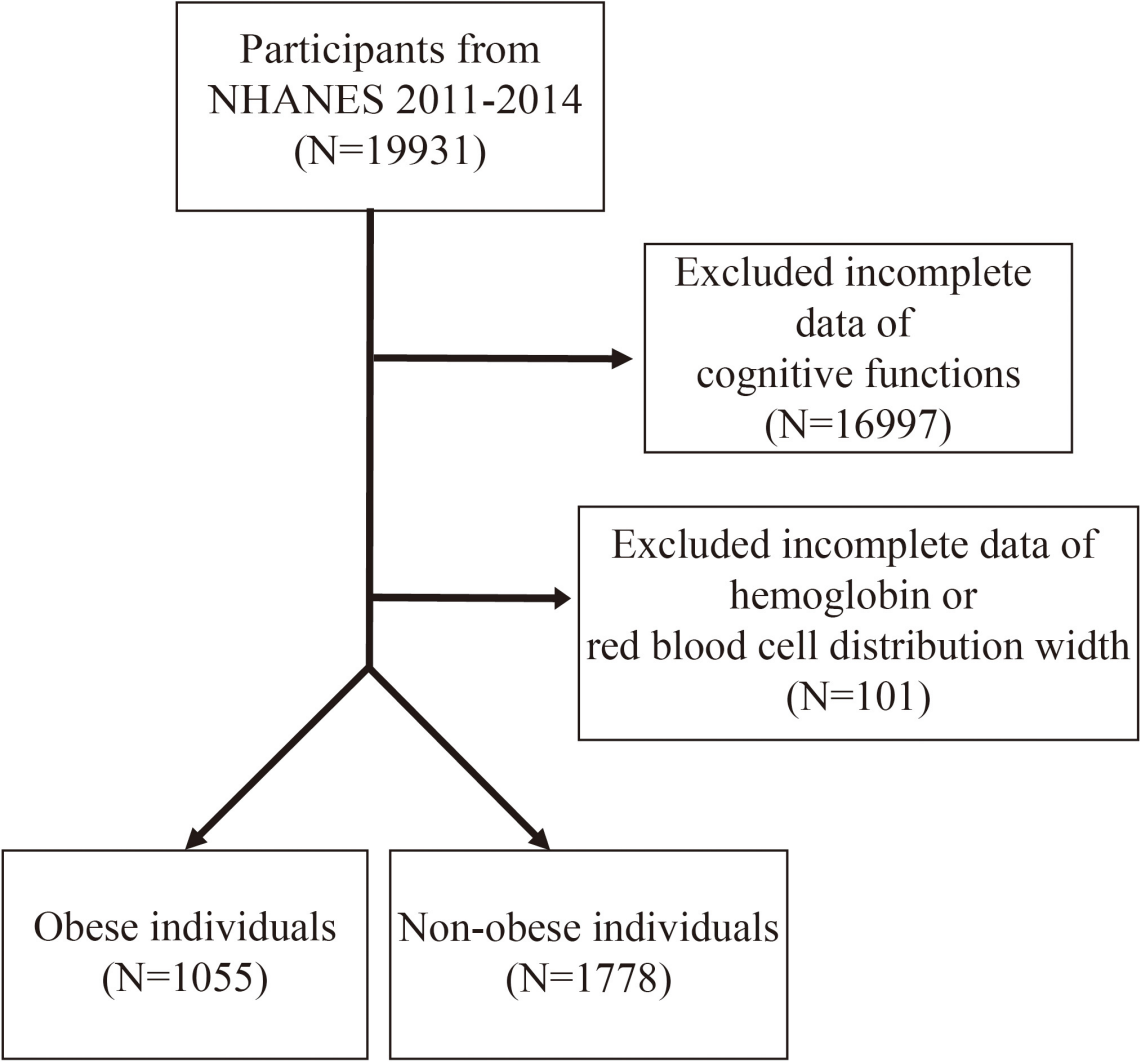
Flow chart of participants selection.

This study focused on the NHANES dataset from 2011 to 2014 (25), with an initial inclusion of 19,931 participants. The NCHS Research Ethics Review Board approved all NHANES protocols of the survey (26). According to the NHANES database criteria, only individuals aged 60 years and above met the basic requirements for cognitive function testing. As shown in Figure 1, the study excluded participants with incomplete cognitive function test data (*n* = 16,997) and those with missing hemoglobin and red blood cell distribution width (RDW) data (*n* = 101). The remaining participants were classified into an obese population [Body mass index (BMI) ≥ 30 kg/m^2^, *n* = 1,055] and non-obese individuals (BMI < 30 kg/m^2^, *n* = 1,778).

### 2.2 HRR calculation

The HRR was calculated based on the ratio of hemoglobin to RDW (21).

#### 2.2.1 Cognitive function assessment

Cognitive function was assessed using the following tests: the word learning and recall modules from the Consortium to Establish a Registry for Alzheimer’s Disease (CERAD), the Animal Fluency Test (AFT), and the Digit Symbol Substitution Test (DSST) (27). Overall cognitive function was assessed using standardized total Z-scores [*Z* = (x−μ)/σ], where x is the specific test score, μ is the mean, and σ is the standard deviation (28). Low cognitive function (LCF) was defined as the lowest quartile of the test scores (19, 29), with Z-scores ≤ 2 indicating low risk of low cognitive function and Z-scores > 2 indicating high risk (High Risk of LCF by Z) (27, 30).

#### 2.2.2 Covariates assessment

The covariates gathered for this study encompassed a range of demographic and health-related factors, including sex, age, race, education level, marital status, poverty-to-income ratio (PIR), BMI, smoking habits, alcohol use, and self-reported medical conditions such as diabetes, hypertension, cardiovascular diseases, and stroke.

### 2.3 Statistical analysis

All analyses were performed using EmpowerStats and R software. Participants were grouped according to HRR quartiles, and *t*-tests and chi-square tests were used to assess continuous and categorical variables, respectively. Multiple regression models were used to evaluate the relationship between HRR, both as a continuous variable and by quartile, and cognitive function. A smooth curve fitting model was applied to explore the relationship between HRR and cognitive test scores, as well as low cognitive function. Additionally, subgroup analyses and interaction analyses were conducted based on stratified factors such as age and sex. A *P*-value of <0.05 was considered statistically significant.

## 3 Results

### 3.1 Baseline characteristics

1,055 obese individuals aged ≥60 years were included, with a mean age of 68.65 ± 6.45 years. Among them, 57.16% were females, and 47.01% were non-Hispanic White. The mean hemoglobin-to-red blood cell distribution width ratio (HRR) was 1.01 ± 0.15. The mean scores for the CERAD, AFT, DSST tests, and the total Z-score were 26.06 ± 6.22, 16.86 ± 5.46, 46.11 ± 17.03, and −0.00 ± 2.40, respectively.

According to the CERAD, AFT, and DSST test scores, the cutoff points for low cognitive function were 22, 13, and 34, respectively. Scores below these cutoff points were considered to indicate low cognitive function (LCF), categorized as LCF by CERAD, LCF by AFT, and LCF by DSST.

Baseline information based on HRR quartiles is presented in Table 1. Table 1 presents the BMI characteristics of the obese population aged ≥60 years stratified by HRR quartiles. The total obese population had a mean BMI of 35.38 ± 5.33 kg/m^2^. Across HRR quartiles, BMI showed a decreasing trend: 36.63 ± 6.63 kg/m^2^ in Q1 (HRR range: 0.44–0.92), 35.74 ± 5.38 kg/m^2^ in Q2 (HRR range: 0.92–1.01), 34.84 ± 4.29 kg/m^2^ in Q3 (HRR range: 1.01–1.10), and 34.32 ± 4.44 kg/m^2^ in Q4 (HRR range: 1.11–1.43). A statistically significant difference in BMI among the HRR quartile groups was observed (*P* < 0.001).

**TABLE 1:** Basic characteristics of participants by hemoglobin-to-red blood cell distribution width ratio among the obese population aged ≥60 years.

Characteristics	Hemoglobin-to-red blood cell distribution width ratio (HRR)					*P*-value
	Total	Q1 (0.44–0.92)	Q2 (0.92–1.01)	Q3 (1.01–1.10)	Q4 (1.11–1.43)	
**N**	1055	264	261	266	264	
**Age (years)**	68.65 ± 6.45	69.30 ± 6.34	68.47 ± 6.47	68.45 ± 6.78	68.38 ± 6.16	0.307
**Sex, (%)**						<0.001
Male	452 (42.84%)	69 (26.14%)	81 (31.03%)	119 (44.74%)	183 (69.32%)	
Female	603 (57.16%)	195 (73.86%)	180 (68.97%)	147 (55.26%)	81 (30.68%)	
**Race, (%)**						<0.001
Mexican American	109 (10.33%)	22 (8.33%)	22 (8.43%)	31 (11.65%)	34 (12.88%)	
Other Hispanic	104 (9.86%)	21 (7.95%)	34 (13.03%)	28 (10.53%)	21 (7.95%)	
Non-Hispanic White	496 (47.01%)	86 (32.58%)	115 (44.06%)	135 (50.75%)	160 (60.61%)	
Non-Hispanic Black	315 (29.86%)	128 (48.48%)	87 (33.33%)	61 (22.93%)	39 (14.77%)	
Other races	31 (2.94%)	7 (2.65%)	3 (1.15%)	11 (4.14%)	10 (3.79%)	
**Education level, (%)**						0.007
Less than high school	277 (26.26%)	76 (28.79%)	71 (27.20%)	78 (29.32%)	52 (19.70%)	
High school or GED	259 (24.55%)	74 (28.03%)	72 (27.59%)	53 (19.92%)	60 (22.73%)	
Above high school	519 (49.19%)	114 (43.18%)	118 (45.21%)	135 (50.75%)	152 (57.58%)	
**Marital status**						<0.001
Married/living with a partner	576 (54.60%)	122 (46.21%)	133 (50.96%)	154 (57.89%)	167 (63.26%)	
Living alone	479 (45.40%)	142 (53.79%)	128 (49.04%)	112 (42.11%)	97 (36.74%)	
**Family PIR**	2.51 ± 1.51	2.34 ± 1.42	2.36 ± 1.50	2.55 ± 1.54	2.79 ± 1.55	0.002
**BMI (kg/m^2^)**	35.38 ± 5.33	36.63 ± 6.63	35.74 ± 5.38	34.84 ± 4.29	34.32 ± 4.44	<0.001
**Smoking habits, (%)**						0.011
Ever	522 (49.48%)	121 (45.83%)	122 (46.74%)	125 (46.99%)	154 (58.33%)	
Never	533 (50.52%)	143 (54.17%)	139 (53.26%)	141 (53.01%)	110 (41.67%)	
**Alcohol use, (%)**						<0.001
Yes	694 (65.78%)	149 (56.44%)	167 (63.98%)	180 (67.67%)	198 (75.00%)	
No	361 (34.22%)	115 (43.56%)	94 (36.02%)	86 (32.33%)	66 (25.00%)	
**Diabetes, (%)**						<0.001
Yes	349 (33.08%)	117 (44.32%)	90 (34.48%)	86 (32.33%)	56 (21.21%)	
No	647 (61.33%)	130 (49.24%)	157 (60.15%)	164 (61.65%)	196 (74.24%)	
Borderline	59 (5.59%)	17 (6.44%)	14 (5.36%)	16 (6.02%)	12 (4.55%)	
**Hypertension, (%)**						<0.001
Yes	768 (72.80%)	217 (82.20%)	191 (73.18%)	188 (70.68%)	172 (65.15%)	
No	287 (27.20%)	47 (17.80%)	70 (26.82%)	78 (29.32%)	92 (34.85%)	
**Heart failure, (%)**						0.002
**Yes**	102 (9.67%)	40 (15.15%)	26 (9.96%)	21 (7.89%)	15 (5.68%)	
**No**	953 (90.33%)	224 (84.85%)	235 (90.04%)	245 (92.11%)	249 (94.32%)	
**Coronary heart disease, (%)**						0.883
Yes	100 (9.48%)	25 (9.47%)	27 (10.34%)	26 (9.77%)	22 (8.33%)	
No	955 (90.52%)	239 (90.53%)	234 (89.66%)	240 (90.23%)	242 (91.67%)	
**Stroke, (%)**						0.027
Yes	78 (7.39%)	30 (11.36%)	19 (7.28%)	16 (6.02%)	13 (4.92%)	
No	977 (92.61%)	234 (88.64%)	242 (92.72%)	250 (93.98%)	251 (95.08%)	
**HB**	13.72 ± 1.46	12.11 ± 0.99	13.34 ± 0.83	14.11 ± 0.76	15.31 ± 0.91	<0.001
**RDW**	13.74 ± 1.18	14.94 ± 1.30	13.76 ± 0.83	13.36 ± 0.68	12.92 ± 0.68	<0.001
**HRR**	1.01 ± 0.15	0.82 ± 0.08	0.97 ± 0.03	1.06 ± 0.03	1.19 ± 0.06	<0.001
**CERAD**	26.06 ± 6.22	25.42 ± 6.14	26.86 ± 6.04	26.26 ± 6.28	25.70 ± 6.36	0.04
**AFT**	16.86 ± 5.46	14.89 ± 4.90	17.35 ± 5.56	17.38 ± 5.42	17.84 ± 5.47	<0.001
**DSST**	46.11 ± 17.03	41.32 ± 16.94	46.37 ± 15.64	47.01 ± 17.10	49.73 ± 17.35	<0.001
**Total z score**	−0.00 ± 2.40	−0.75 ± 2.29	0.23 ± 2.35	0.18 ± 2.38	0.33 ± 2.43	<0.001

Mean ± SD for continuous variables: the *P*-value was calculated by the weighted linear regression model; (%) for categorical variables: the *P*-value was calculated by the weighted chi-square test. N, number; PIR, the ratio of income to poverty, BMI, body mass index; Q, quartile; HRR, hemoglobin-to-red blood cell distribution width ratio; CERAD, the Consortium to Establish a Registry for Alzheimer’s Disease test; AFT, the Animal Fluency Test; DSST, the Digit Symbol Substitution Test.

### 3.2 Association between HRR and cognitive function

Table 2 and Table 3 collectively illustrate the association between the HRR and cognitive function, including cognitive test scores and low cognitive function, among obese individuals aged ≥60 years across three regression models (Model 1: unadjusted; Model 2: adjusted for age, sex, and race; Model 3: fully adjusted for demographic, lifestyle, and comorbidity factors).

**TABLE 2:** Association between HRR and cognitive test scores among the obese population.

HRR	CERAD		AFT		DSST		Total Z score	
	β (95% CI)	*P*-value	β (95% CI)	*P*-value	β (95% CI)	*P*-value	β (95% CI)	*P*-value
** Model 1**								
Continuous	2.55 (−1.02, 6.12)	0.1713	6.85 (3.53, 10.17)	0.0003	26.54 (17.26, 35.81)	<0.0001	3.22 (2.02, 4.42)	<0.0001
** Categories**								
Quartile 1	Reference		Reference		Reference		Reference	
Quartile 2	1.74 (0.01, 3.46)	0.0583	2.67 (1.21, 4.12)	0.0012	5.07 (1.17, 8.97)	0.0163	1.07 (0.41, 1.72)	0.0034
Quartile 3	1.29 (−0.17, 2.75)	0.0947	2.69 (1.38, 4.01)	0.0004	6.63 (2.90, 10.37)	0.0016	1.09 (0.55, 1.63)	0.0005
Quartile 4	1.38 (−0.32, 3.07)	0.1222	3.06 (1.80, 4.32)	<0.0001	10.08 (5.83, 14.33)	0.0001	1.37 (0.80, 1.95)	0.0001
P for trend		0.2412		0.0002		<0.0001		0.0001
** Model 2**								
Continuous	3.28 (−0.76, 7.31)	0.1244	3.54 (−0.27, 7.35)	0.0806	21.75 (14.74, 28.76)	<0.0001	2.45 (1.19, 3.71)	0.0008
** Categories**								
Quartile 1	Reference		Reference		Reference		Reference	
Quartile 2	1.68 (0.06, 3.30)	0.0535	2.07 (0.70, 3.43)	0.0068	3.31 (−0.18, 6.81)	0.0755	0.84 (0.26, 1.42)	0.0092
Quartile 3	1.31 (−0.17, 2.80)	0.0952	1.70 (0.47, 2.94)	0.0126	4.38 (0.94, 7.81)	0.0201	0.78 (0.26, 1.30)	0.0076
Quartile 4	1.71 (0.00, 3.42)	0.0620	1.80 (0.34, 3.27)	0.0243	8.17 (4.88, 11.45)	0.0001	1.08 (0.54, 1.63)	0.0007
P for trend		0.1318		0.0733		<0.0001		0.0014
** Model 3**								
Continuous	1.75 (−2.53, 6.03)	0.4413	2.21 (−0.69, 5.10)	0.1664	14.45 (7.55, 21.35)	0.0021	1.53 (0.40, 2.67)	0.0240
** Categories**								
Quartile 1	Reference		Reference		Reference		Reference	
Quartile 2	1.61 (0.11, 3.10)	0.0679	2.25 (1.00, 3.50)	0.0078	3.56 (0.71, 6.40)	0.0402	0.88 (0.35, 1.41)	0.0112
Quartile3	1.10 (−0.29, 2.49)	0.1597	1.56 (0.48, 2.64)	0.0223	3.37 (0.56, 6.19)	0.0469	0.66 (0.22, 1.10)	0.0180
Quartile 4	1.19 (−0.43, 2.81)	0.1889	1.37 (0.27, 2.46)	0.0403	5.75 (2.69, 8.81)	0.0062	0.78 (0.32, 1.24)	0.0104
P for trend		0.3671		0.1625		0.0055		0.0215

Model 1: no covariates were adjusted. Model 2: age, sex, and race were adjusted. Model 3: age, sex, race, education level, marital status, poverty-to-income ratio (PIR), body mass index (BMI), smoking habits, alcohol use, diabetes, hypertension, cardiovascular diseases, and stroke. Q, quartile; HRR, hemoglobin-to-red blood cell distribution width ratio; CERAD, the Consortium to Establish a Registry for Alzheimer’s Disease test; AFT, the Animal Fluency Test; DSST, the Digit Symbol Substitution Test.

**TABLE 3:** Association between HRR and low cognitive function among the obese population.

HRR	LCF by CERAD		LCF by AFT		LCF by DSST		High risk of LCF by Z	
	OR (95% CI)	*P*-value	OR (95% CI)	*P*-value	OR (95% CI)	*P*-value	OR (95% CI)	*P*-value
** Model 1**								
Continuous	0.28 (0.11, 0.73)	0.0137	0.07 (0.01, 0.38)	0.0046	0.02 (0.01, 0.06)	<0.0001	5.26 (1.63, 16.97)	0.0093
** Categories**								
Quartile 1	Reference		Reference		Reference		Reference	
Quartile 2	0.68 (0.38, 1.23)	0.2153	0.44 (0.26, 0.74)	0.0048	0.48 (0.30, 0.78)	0.0057	2.18 (0.99, 4.78)	0.0616
Quartile 3	0.58 (0.37, 0.91)	0.0236	0.44 (0.29, 0.67)	0.0007	0.43 (0.25, 0.73)	0.0047	2.08 (1.00, 4.33)	0.0595
Quartile 4	0.55 (0.35, 0.89)	0.0210	0.34 (0.18, 0.63)	0.0018	0.23 (0.16, 0.35)	<0.0001	2.11 (1.10, 4.06)	0.0335
P for trend		0.0289		0.0037		<0.0001		0.0342
** Model 2**								
Continuous	0.22 (0.08, 0.66)	0.0115	0.17 (0.03, 1.03)	0.0654	0.03 (0.01, 0.13)	0.0001	3.15 (0.68, 14.71)	0.1563
** Categories**								
Quartile 1	Reference		Reference		Reference		Reference	
Quartile 2	0.66 (0.34, 1.28)	0.2310	0.51 (0.27, 0.95)	0.0458	0.53 (0.28, 1.00)	0.0628	2.02 (0.88, 4.61)	0.1099
Quartile 3	0.55 (0.34, 0.89)	0.0243	0.57 (0.37, 0.89)	0.0215	0.50 (0.26, 0.96)	0.0485	1.72 (0.77, 3.82)	0.1986
Quartile 4	0.47 (0.27, 0.82)	0.0134	0.48 (0.24, 0.96)	0.0508	0.26 (0.14, 0.46)	0.0001	1.72 (0.84, 3.52)	0.1509
P for trend		0.0202		0.0704		0.0007		0.2708
** Model 3**								
Continuous	0.43 (0.13, 1.39)	0.1887	0.22 (0.04, 1.22)	0.1148	0.04 (0.01, 0.23)	0.0043	1.48 (0.28, 7.75)	0.6540
** Categories**								
Quartile 1	Reference		Reference		Reference		Reference	
Quartile 2	0.66 (0.34, 1.29)	0.2592	0.48 (0.26, 0.91)	0.0531	0.48 (0.22, 1.06)	0.1057	2.22 (0.95, 5.20)	0.1028
Quartile 3	0.56 (0.33, 0.94)	0.0600	0.59 (0.39, 0.88)	0.0323	0.45 (0.22, 0.89)	0.0520	1.54 (0.71, 3.35)	0.3092
Quartile 4	0.57 (0.33, 0.98)	0.0761	0.52 (0.28, 0.98)	0.0766	0.29 (0.14, 0.58)	0.0081	1.35 (0.63, 2.87)	0.4579
P for trend		0.0813		0.1162		0.0107		0.9736

Model 1: no covariates were adjusted. Model 2: age, sex, and race were adjusted. Model 3: age, sex, race, education level, marital status, poverty-to-income ratio (PIR), body mass index (BMI), smoking habits, alcohol use, diabetes, hypertension, cardiovascular diseases, and stroke. Q, quartile; HRR, hemoglobin-to-red blood cell distribution width ratio; CERAD, the Consortium to Establish a Registry for Alzheimer’s Disease test; AFT, the Animal Fluency Test; DSST, the Digit Symbol Substitution Test. LCF, low cognitive function.

For the CERAD test, Table 2 showed no significant association between HRR and CERAD scores in any model (all *P* > 0.05). Consistently, Table 3 revealed that the initial inverse association between HRR and CERAD-defined LCF in Model 1 (continuous HRR: OR = 0.28, 95% CI, 0.11–0.73, *P* = 0.0137) and Model 2 (continuous HRR: OR = 0.22, 95% CI, 0.08–0.66, *P* = 0.0115) weakened in Model 3, with no significant correlation (continuous HRR: OR = 0.43, 95% CI, 0.13–1.39, *P* = 0.1887; P for trend = 0.0813).

Regarding the AFT, Table 2 indicated a significant positive correlation between HRR and AFT scores in Model 1 (continuous HRR: β = 6.85, 95% CI, 3.53–10.17, *P* = 0.0003), which weakened after adjustment, becoming non-significant in Model 3 (β = 2.21, 95% CI, −0.69 to 5.10, *P* = 0.1664). Parallelly, Table 3 showed that the initial inverse association between HRR and AFT-defined LCF in Model 1 (continuous HRR: OR = 0.07, 95% CI, 0.01–0.38, *P* = 0.0046) was attenuated in Model 3, with no significant association (continuous HRR: OR = 0.22, 95% CI, 0.04–1.22, *P* = 0.1148; P for trend = 0.1162).

For the DSST, both tables demonstrated consistent and robust associations. Table 2 showed a significant positive correlation between HRR and DSST scores in Model 3 (continuous HRR: β = 14.45, 95% CI, 7.55–21.35, *P* = 0.0021; quartiles 2–4 vs. quartile 1: all *P* < 0.05; P for trend = 0.0055). Correspondingly, Table 3 revealed a significant inverse association between HRR and DSST-defined LCF in Model 3 (continuous HRR: OR = 0.04, 95% CI, 0.01–0.23, *P* = 0.0043; quartile 4 vs. quartile 1: OR = 0.29, 95% CI, 0.14–0.58, *P* = 0.0081; P for trend = 0.0107).

With respect to total cognitive Z-scores, Table 2 found a significant positive association with HRR in Model 3 (continuous HRR: β = 1.53, 95% CI, 0.40–2.67, *P* = 0.0240; quartiles 2–4 vs. quartile 1: all *P* < 0.05; P for trend = 0.0215). In contrast, Table 3 showed that the initial positive association between HRR and high risk of LCF by Z in Model 1 (continuous HRR: OR = 5.26, 95% CI, 1.63–16.97, *P* = 0.0093) was attenuated in Model 3, with no significant correlation (continuous HRR: OR = 1.48, 95% CI, 0.28–7.75, *P* = 0.6540; P for trend = 0.9736).

Overall, these results indicate that HRR is stably associated with DSST-related cognitive performance in obese population, showing a positive correlation with DSST scores and an inverse correlation with DSST-defined LCF in fully adjusted models, while its associations with CERAD, AFT, and high risk of LCF by total cognitive Z scores are not robust after comprehensive covariate adjustment.

Incidentally, We have conducted an analysis of the association between HRR and cognition in the non-obese (BMI < 30 kg/m^2^) populations. In Model 3, adjusted for covariates, there was no significant association between HRR and cognition in the non-obese population. Specifically, no significant correlation was found between HRR and CERAD, AFT, DSST, or total Z score. The results were as follows: HRR and CERAD (β = −1.95; 95% CI, −4.46 to 0.55), HRR and AFT (β = 1.16; 95% CI, −0.24 to 2.56), HRR and DSST (β = 3.30; 95% CI, −2.22 to 8.83), HRR and total Z score (β = 0.10; 95% CI, −0.57 to 0.76). Additionally, there was no significant association between HRR and LCF or high-risk groups. The specific results were: HRR and LCF by CERAD (OR = 1.69; 95% CI, 0.62–4.59), HRR and LCF by AFT (OR = 0.83; 95% CI, 0.34–2.03), HRR and LCF by DSST (OR = 0.40; 95% CI, 0.05–3.08), HRR and High Risk by Z (OR = 0.71; 95% CI, 0.22–2.32).

**FIGURE 2:**
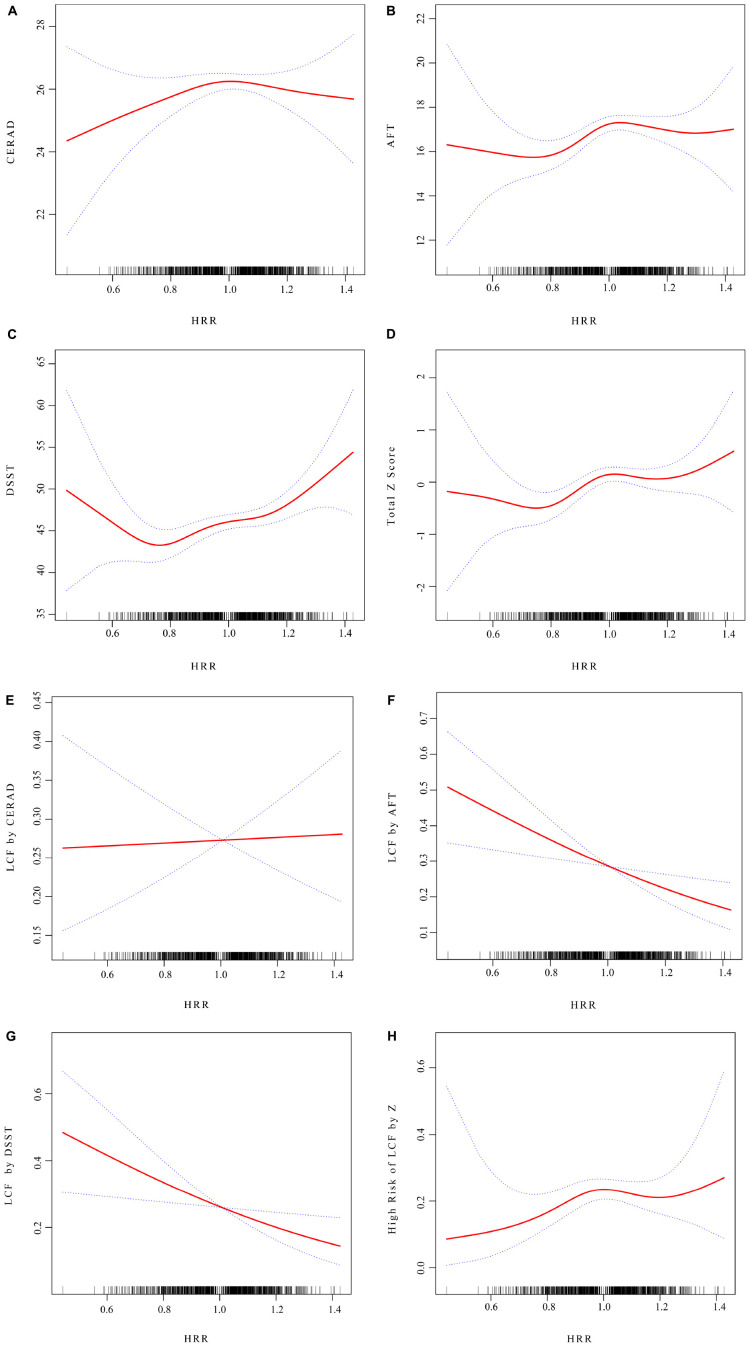
The non-linear associations between HRR and cognitive function. The solid red line represents the smooth curve fit between variables. Blue bands represent the 95% of confidence interval from the fit. **(A)** HRR and CERAD score; **(B)** HRR and AFT score; **(C)** HRR and DSST score; **(D)** HRR and total Z score; **(E)** HRR and LCF by CERAD; **(F)** HRR and LCF by AFT; **(G)** HRR and LCF by DSST; **(H)** HRR and High Risk of LCF by Z.

The smooth curve for HRR and CERAD scores showed a turning point at HRR = 0.94 (Figure 2A and Table 4). To the left of this point, HRR was positively correlated with CERAD scores (β = 6.43; 95% CI, 0.79–12.06; *P* = 0.0256). The smooth curve for HRR and AFT scores showed a turning point at HRR = 1.01 (Figure 2B and Table 4). To the left of this point, HRR was positively correlated with AFT scores (β = 6.11; 95% CI, 2.40–9.82; *P* = 0.0013). The smooth curve for HRR and DSST scores showed a turning point at HRR = 0.75 (Figure 2C and Table 4). To the right of this point, HRR was positively correlated with DSST scores (β = 13.22; 95% CI, 6.80–19.63; *P* < 0.0001). HRR showed a linear relationship with total Z-scores (LLR = 0.056) and was positively correlated with total Z-scores (β = 1.16; 95% CI, 0.28–2.05; *P* = 0.0101) (Figure 2D and Table 4). For low cognitive function: HRR did not show relationship with LCF by CERAD (Figure 2E and Table 4). HRR showed a linear relationship with LCF by AFT (LLR = 0.5), and in the LCF by AFT group, HRR was negatively correlated with the occurrence of low cognitive function (OR = 0.18; 95% CI, 0.06–0.57; *P* = 0.0035) (Figure 2F and Table 5). HRR showed a linear relationship with LCF by DSST (LLR = 0.324), and in the LCF by DSST group, HRR was negatively correlated with the occurrence of low cognitive function (OR = 0.17; 95% CI, 0.05–0.67; *P* = 0.0109) (Figure 2G and Table 5). In the High Risk of LCF by Z group, the smooth curve showed a positive correlation between HRR and high-risk low cognitive function, with a turning point at HRR = 0.94 (OR = 55.53; 95% CI, 1.47–2097.52; *P* = 0.0302) (Figure 2H and Table 5).

**TABLE 4:** Threshold effect analysis of HRR on cognitive test scores using a two-segment linear regression model among the obese population.

	CERAD		AFT		DSST		Total Z score	
	β (95% CI)	*P*-value	β (95% CI)	*P*-value	β (95% CI)	*P*-value	β (95% CI)	*P*-value
Linear effect model	0.81 (−1.86, 3.48)	0.5509	2.52 (0.20, 4.84)	0.0333	9.70 (3.91, 15.49)	0.0011	1.16 (0.28, 2.05)	0.0101
** Non-linear model**								
Inflection point (K)	0.94		1.01		0.75		1.00	
<K-segment effect	6.43 (0.79, 12.06)	0.0256	6.11 (2.40, 9.82)	0.0013	−39.09 (−78.29, 0.11)	0.0509	2.29 (0.82, 3.76)	0.0023
>K-segment effect	−2.73 (−6.85, 1.38)	0.1934	−2.14 (−6.58, 2.29)	0.3433	13.22 (6.80, 19.63)	<0.0001	−0.16 (−1.79, 1.47)	0.8500
Log likelihood ratio	0.025		0.014		0.013		0.056	

HRR, hemoglobin-to-red blood cell distribution width ratio; CERAD, the Consortium to Establish a Registry for Alzheimer’s Disease test; AFT, the Animal Fluency Test; DSST, the Digit Symbol Substitution Test.

**TABLE 5:** Threshold effect analysis of HRR on low cognitive function using a two-segment linear regression model among the obese population.

	LCF by CERAD		LCF by AFT		LCF by DSST		High risk of LCF by Z	
	OR (95% CI)	*P*-value	OR (95% CI)	*P*-value	OR (95% CI)	*P*-value	OR (95% CI)	*P*-value
Linear effect model	1.10 (0.34, 3.51)	0.8769	0.18 (0.06, 0.57)	0.0035	0.17 (0.05, 0.67)	0.0109	2.11 (0.53, 8.44)	0.2930
** Non-linear model**								
Inflection point (K)	0.75		0.81		0.75		0.94	
<K-segment effect	432.38 (0.15, 1218565.73)	0.1343	0.88 (0.01, 96.00)	0.9589	15.97 (0.00, 135904.47)	0.5484	55.53 (1.47, 2097.52)	0.0302
>K-segment effect	0.73 (0.20, 2.63)	0.6246	0.14 (0.03, 0.57)	0.0060	0.12 (0.03, 0.56)	0.0068	0.45 (0.06, 3.55)	0.4491
Log likelihood ratio	0.135		0.5		0.324		0.042	

HRR, hemoglobin-to-red blood cell distribution width ratio; CERAD, the Consortium to Establish a Registry for Alzheimer’s Disease test; AFT, the Animal Fluency Test; DSST, the Digit Symbol Substitution Test. LCF, low cognitive function.

### 3.3 Subgroup analyses

**FIGURE 3:**
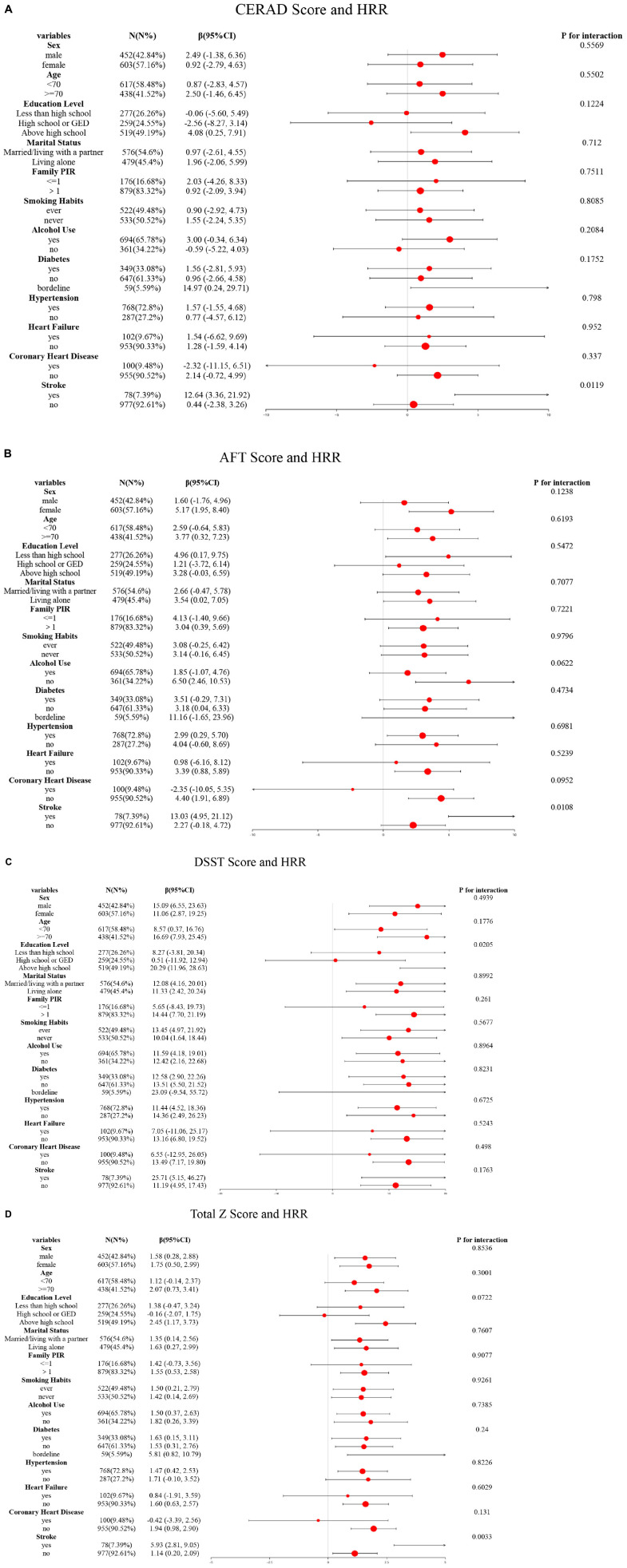
Subgroup logistic regression analysis for the association between HRR and cognitive scores. **(A)** HRR and CERAD score; **(B)** HRR and AFT score; **(C)** HRR and DSST score; **(D)** HRR and total Z score.

To further assess the effect of HRR in the obese population on cognitive test scores and low cognitive function, stratified analyses were performed based on age, sex, education level, smoking, alcohol consumption, family poverty-to-income ratio, diabetes, and cardiovascular diseases as covariates. The obese population consisted of 1,055 participants (452 men and 603 women), including 617 individuals aged <70 years and 438 aged ≥70 years. In terms of education level, 277 had less than high school education, 259 had high school or GED, and 519 had more than high school education. Regarding family poverty-to-income ratio, 176 had a ratio ≤1, and 879 had a ratio >1. Among them, 576 were married or living with parents, while 479 lived alone; additionally, 522 had a history of smoking, 533 had never smoked, 694 had a history of alcohol consumption, and 361 had no alcohol consumption. The population included 349 with diabetes, 647 without diabetes, and 59 with borderline glucose tolerance; 768 had hypertension, 287 did not; 102 had heart failure, 953 did not; 100 had coronary heart disease, 955 did not; and 78 had a history of stroke, 977 did not. In the stroke subgroup, HRR showed a stronger correlation with CERAD scores (*P* = 0.0119) (Figure 3A), AFT scores (*P* = 0.0108) (Figure 3B), and total Z-scores (*P* = 0.0033) (Figure 3D). In the high-education group, HRR was more strongly correlated with DSST scores (*P* = 0.0205) (Figure 3C).

## 4 Discussion

The study results show that in obese individuals, higher HRR is associated with better DSST performance and reduced likelihood of scoring below the cognitive impairment threshold. The results of the study suggest that HRR may have a positive effect on certain cognitive functions, particularly in areas like reaction time, attention, and working memory. This highlights the potential of HRR as a biomarker for cognitive function and a tool for identifying individuals at risk for cognitive decline.

With the increase of age, obvious declines occur in multiple specific cognitive domains: processing speed continues to decline, affecting the performance of language fluency and other aspects; complex attention tasks such as selective attention and divided attention decline significantly, and working memory is affected by the slowdown of information processing; episodic memory and semantic memory decline; visual naming and verbal fluency decrease; visual construction ability reduces; abilities such as concept formation, abstract reasoning, mental flexibility and response inhibition in executive function decline (31, 32). The neurochemical properties and anatomical structure of the brain exhibit cumulative changes with age, with a significant decline in dopaminergic neuromodulation (32). The volume of gray and white matter gradually decreases, and the atrophy of polymodal cortical regions is particularly prominent, while the atrophy process of the hippocampus is accelerated by vascular factors (32). There are individual differences in the senescent changes of brain structure, especially the significant differences in the degree of age-related contraction in regions such as the lateral prefrontal cortex, prefrontal white matter, and hippocampus (32).

This study found that the protective effect of HRR on cognitive function was more pronounced in individuals with higher educational attainment, a result that can be theoretically explained by the mediating mechanism of cognitive reserve (33). In studies by Clare et al., educational level has been explicitly identified as a core component of cognitive reserve (33). Cognitive reserve buffers the impact of neuropathological changes on cognitive function by optimizing brain network recruitment strategies or activating alternative cognitive pathways (34). Higher educational attainment is often associated with greater cognitive reserve, which amplifies the protective effects of physiological processes reflected by HRR–such as oxygen supply status and inflammation–on cognition through enhancing neurovascular coupling efficiency or metabolic compensatory capacity (35).

In our study, a positive association between higher HRR and better cognitive performance was observed in obese individuals, but no such relationship was found in non-obese counterparts. This discrepancy may be attributed to the role of obesity in cognitive decline. Obesity is closely linked to various chronic diseases, and its negative impact on the brain is increasingly gaining attention (36). Obesity affects brain function and leads to cognitive decline through mechanisms such as neuroinflammation, oxidative stress, and alterations in the gut-brain axis (9, 36, 37). Anfal Al-Dalaeen et al. pointed out that neuroinflammation, oxidative stress, and reduced local blood flow induced by obesity jointly affect the brain (36). These factors disrupt the metabolic functions of the hypothalamus and the hippocampus, ultimately leading to cognitive impairment (36, 38). Alyson A. Miller et al. further emphasized that systemic inflammation and increased free fatty acids caused by obesity can lead to local inflammation in the hypothalamus, which then affects cognitive-related brain regions, such as the hippocampus and amygdala, thereby exacerbating cognitive decline (38, 39). Sarah-Jane Leigh et al. reviewed the relationship between obesity, high-fat diets, and cognitive impairment, finding that changes in the gut microbiome, systemic and central nervous system inflammation, and alterations in the blood-brain barrier are key mechanisms in this process (10, 37).

In our study, Model 3–adjusted for multiple covariates–still showed that higher HRR was positively associated with better cognitive performance, including higher DSST scores and reduced low cognitive function assessed by DSST, suggesting HRR could act as a biomarker for cognitive function in obese individuals. An increasing number of studies have shown a significant association between hemoglobin levels and cognitive function (40–44). Low hemoglobin levels have been identified as a potential risk factor for cognitive decline (42–48). Yi-Xuan Qiang et al. revealed that anemia is connected to a risk increase of more than 50% for all-cause dementia, with brain structure changes being a potential contributing factor that affects cognitive abilities (49). Laura M. Winchester et al. pointed out that lower hemoglobin levels were significantly associated with cognitive decline, particularly in the domains of reaction time and reasoning abilities (48). Andrea L. C. Schneider et al. also found that lower hemoglobin levels were negatively correlated with cognitive domains such as processing speed, attention, and working memory, as assessed by the DSST (16). In patients suffering from stroke, lower hemoglobin levels have been correlated with a higher likelihood of post-stroke cognitive impairment (50). Some studies have also found that higher hemoglobin levels in stroke patients are positively correlated with the maintenance of cognitive function (51). However, Raj C. Shah et al. highlighted that both very low and very high hemoglobin levels were associated with lower cognitive function, particularly in areas such as semantic memory and perceptual speed (52, 53). This suggests that hemoglobin may have a bidirectional effect on cognitive function. Despite these findings, some studies have not found a direct relationship between hemoglobin levels and cognitive function (16, 54, 55). Beydoun et al. found that in individuals with anemia, no significant association was observed between RDW and cognitive performance (54). By contrast, in non-anemic populations, the association between RDW and cognition was more consistent (54). This lack of association with anemia may be attributed to the elevated RDW in anemic individuals within the sample, which potentially masked the independent effect of hemoglobin (54). Chen et al.’s study recruited healthy old men from Taiwan, China, and used the Cognitive Abilities Screening Instrument Chinese version and the Wechsler Digit Span Task test for cognitive assessment (55). The study’s conclusions indicated that the association between hemoglobin levels and cognitive function was influenced by differences in study populations and cognitive assessment methods (55). Schneider et al. found in their study on different hemoglobin concentrations that the sample size of individuals with high hemoglobin was relatively small (21 men and 56 women), a limitation that compromised the statistical power of analyses examining associations between this group and cognitive function (16). In the prospective follow-up with a mean duration of 6 years, the study further revealed no significant associations between overall anemia or its subtypes and declines in cognitive function (16). This result may be attributed to multiple factors: the relatively young baseline age of the study participants (mean age 57 years), the relatively short follow-up period, and the presence of attrition bias–those who were lost to follow-up were predominantly older individuals with lower educational attainment and multiple vascular risk factors (16).

The influence of hemoglobin on cognitive function may be related to several mechanisms, such as chronic hypoxia, β-amyloid deposition, and neuroinflammation (52, 56). When hemoglobin levels are too low, cerebral blood flow cannot meet the oxygen demands, leading to impaired brain cell function and exacerbating cognitive decline (16, 17). Additionally, a reduction in erythropoietin receptor expression may worsen neural damage, further increasing the risk of cognitive decline (52). Different types of anemia may affect brain function through different mechanisms. For example, iron-deficiency anemia may impair cognitive function by interfering with key enzymes in brain cell metabolism (57), while vitamin B12 and folate deficiencies may exacerbate cognitive impairment by affecting the metabolism of homocysteine and acetylcholine (58).

As a core component of HRR, RDW serves as an indicator of red blood cell volume heterogeneity (18). Studies have demonstrated that RDW is associated with inflammation, oxidative stress, and other factors, which play critical roles in cognitive decline (18, 19). Elevated RDW has been associated with various health conditions, and studies suggest it may also serve as a marker for cognitive dysfunction (54, 59, 60). Yi-Xuan Qiang et al. identified a link between RDW levels and the risk of developing Alzheimer’s disease, suggesting that RDW could serve as a valuable biomarker for monitoring cognitive decline (49). Laura M. Winchester et al. found that lower RDW was associated with poorer language reasoning and memory abilities (48). Kyoung Min Kim et al. pointed out that individuals with higher RDW had worse cognitive function and slower gait (61).

The relationship between increased RDW and cognitive decline may be closely related to inflammatory responses (60). Chronic inflammation is one of the key mechanisms of cognitive decline. Systemic inflammation triggers amyloid deposition, activates microglia and astrocytes in the central nervous system, leading to neuroinflammation that damages neuronal structure and function, ultimately affecting cognitive function (62). Yuan Fang et al. found that peripheral inflammation, by disrupting the blood-brain barrier, activates inflammatory responses in the nervous system, further exacerbating cognitive impairment (59, 63).

In summary, an increase in HRR reflects the body’s ability to resist factors such as systemic inflammation and oxidative stress. These mechanisms, acting together, contribute to the protection of cognitive function.

## 5 Strengths and limitations

This study provides evidence of HRR as a potential biomarker for cognitive health in obese populations. However, due to the cross-sectional design, longitudinal clinical trials are needed to further clarify the causal relationship. While multi-ethnic groups were included, Southeast Asian and other Asian populations were underrepresented. Using BMI to classify obesity has inherent limitations: it relies solely on height and weight, failing to distinguish muscle from fat, and cannot reflect true fat distribution in metabolically obese but normal weight individuals. BMI obesity thresholds also vary by ethnicity. Moreover, the failure to comprehensively assess obesity by integrating multi-dimensional indicators such as body fat percentage, waist circumference, and visceral fat area weakens the accuracy and generalizability of the research conclusions (64, 65).

## 6 Conclusion

This study suggest that HRR may serve as a potential biomarker for reflecting cognitive function status: individuals with higher HRR levels demonstrated better performance in cognitive assessments. This discovery indicates that maintaining a higher HRR could be a potential intervention strategy for protecting the cognitive abilities of obese populations. However, this conclusion still requires further validation through longitudinal studies with larger sample sizes, multicenter clinical trials, and exploration of action mechanisms to clarify the clinical application value of HRR as a target for cognitive protection.

## Data Availability

The raw data supporting the conclusions of this article will be made available by the authors, without undue reservation.
